# Supplementary material for: Fourier Ptychographic Microscopy via Alternating Direction Method of Multipliers
Source: Cells. 2022 Apr 30;11(9):1512. doi: 10.3390/cells11091512 (PMC9104836; doi:10.3390/cells11091512)
Supplement: Supplementary file 1 [file cells-11-01512-s001.zip › cells-1668639-supplementary.pdf]

cells MDPI

Article

Fourier ptychographic microscopy via alternating direction method of multipliers: supplemental document (Supplementary)

Supplementary File S1. Proof of iteration equations in ADMM solution

The ADMM method is intended to solve optimization problems in the general form of:

$$\textbf{minimize} \quad f(x)+g(z)$$

$$\textbf{subject to} \quad Ax+Bz=c \tag{S1}$$

where $x\in R^n$ and $z\in R^m$, where $A\in R^{p\times n}$, $B\in R^{p\times m}$ and $c\in R^p$. Function f and g are assumed convex. We formulate the corresponding augmented Lagrangian:

$$L_\rho(x,z,y)=f(x)+g(z)+y^T(Ax+Bz-c)+\frac{\rho}{2}\|Ax+Bz-c\|_2^2 \tag{S2}$$

where ρ is the penalty parameter. By introducing a new variable defined as $u = y/\rho$, the scaled form of x-update in ADMM iterations can be written as:

$$x^+=\arg\min_x\left(f(x)+\frac{\rho}{2}\|Ax-v\|_2^2\right) \tag{S3}$$

where $v = -Bz+c-u$ and the superscript + denotes the next iteration. Here we simply consider the case A=**I**, which is frequently seen in practical application. The righthand side is termed as the proximity operator [41] of f with penalty ρ and denoted

$$\mathbf{prox}_f(v;\rho):=\arg\min_x\left(f(x)+\frac{\rho}{2}\|Ax-v\|_2^2\right) \tag{S4}$$

If $f(x)$ is second-order differentiable, it can be shown that:

$$\mathbf{prox}_f(v;\rho)\approx v-\left[\nabla^2 f(v)+\rho\mathbf{I}\right]^{-1}\nabla f(x) \tag{S5}$$

This is known as Levenberg-Marquardt update, a combination of Gauss Newton and gradient descent to solve nonlinear least square optimization problems. Equation (12) can be expressed as $q_j^{k+1} = \mathbf{prox}_f\ (diag(p)\mathbf{Q}_j s^k-\omega_j^k;\ \alpha)$ and accordingly we can approximate it to:

$$q_j^{k+1}\approx diag(p)\mathbf{Q}_j s^k-\omega_j^k-\left[\frac{\partial^2}{\partial q^2}f_j(q)|_{q=diag(p)\mathbf{Q}_j s^k-\omega_j^k}+\alpha\mathbf{I}\right]^{-1}\frac{\partial}{\partial q}f_j(q)|_{q=diag(p)\mathbf{Q}_j s^k-\omega_j^k} \tag{S6}$$

The first-order derivative of $f_j(q)$ with respect to q is given as:

$$\frac{\partial}{\partial q}f_j(q)=-\mathbf{Q}_j^H\left[\mathcal{F}diag\left(\frac{\sqrt{I_j}}{|F^H q|}\right)\mathcal{F}^H q-\mathcal{F}^H q\right] \tag{S7}$$

The second-order derivative can be approximated by dropping all off-diagonal terms of the matrix $\frac{\partial^2}{\partial q^2}f_j(q)$. Hence, the inversion of Tikhonov-regularized Hessian term can be estimated as:

$$\left[\frac{\partial^2}{\partial q^2} f_j\left(q\right)+\alpha\mathbf{I}\right]^{-1} \approx \mathbf{Q}_j^H diag\left(\frac{1}{1+\alpha}\right)\mathbf{Q}_j \tag{S8}$$

Substitute back to Eq. (S6), and we get Eq. (16).

The characteristic equation of the minimizer for update equation (9) is given as:

$$\sum_{j=1}^{N}\mathbf{Q}_j^H diag\left(\bar{p}\right)\lambda_j^k+\alpha\mathbf{Q}_j^H diag\left(\bar{p}\right)\left(diag\left(p\right)\mathbf{Q}_j s^{k+1}-q_j^{k+1}\right)+\gamma\left(s^{k+1}-\delta\right)=0 \tag{S9}$$

Solve the equation above, and we get Eq. (13).

The subproblem (14) does not have a trivial solution, so we solve it by locating the proximal with respect to p^k:

$$p^{k+1}=\arg\min_p\left\|q_j^{k+1}-diag\left(p\right)Q_j s^{k+1}+\omega_j^k\right\|_2^2+\beta\left\|p-p^k\right\|_2^2 \tag{S10}$$

Since the operation $diag(x)y$ is commutative, the equation above is equivalent to:

$$p^{k+1}=\arg\min_p\left\|q_j^{k+1}-diag\left(Q_j s^{k+1}\right)p+\omega_j^k\right\|_2^2+\beta\left\|p-p^k\right\|_2^2 \tag{S11}$$

We follow the derivation of (16) via proximity operator and obtain Eq. (17), the specific procedure is not detailed here.

Supplementary File S2. Choices of parameters in ADMM solution

Table 2 directly presents the suggested ranges of parameters in ADMM method. In this section, we will discuss how these parameters are chosen elaborately at length. We first give a summarized explanation for their mathematical significance: 1) α is the penalty parameter which measures the extent to which the local auxiliary variables should fit the global variable. 2) β is the regularized parameter applied to stabilize pupil recovery and prevent extreme values of pupil amplitude component. 3) η is the step size which determines how fast the local solutions get close to the global solution. 4) γ is a parameter involved in the regularization term to stabilize optimization, especially for weak-phase objects.

For each parameter, we select three typical values and examine how their variation affects the reconstruction results. Here, we mainly consider two cases: noise-free and 50% Gaussian noise, corresponding to Fig. S1 and Fig. S2 respectively. The results of Poisson noise is quite similar with Gaussian noise and will not detailed here. By contrast, we intuitively conclude that the penalty parameter α and step size η play the leading role in affecting noise tolerance performance. In the presence of Gaussian noise, it can be seen that the curve α=1 generates fluctuation to some degree after about five iterations whether amplitude or phase, while the other two curves remain similarly stable. For step size η, the curve with larger value is characterized by a larger slope during the increase stage and hence reach convergence with a fewer number of iterations, but the difference is small in terms of both iteration numbers and numerical results. However, the two curves with smaller values tend to fluctuate dramatically under the condition of Gaussian noise.

Regarding regularized parameters β and γ, we see no distinct effects that they impose on the noise tolerance performance of ADMM method. Their curves under noise condition seems to simply derive from the downward displacement of those in the noiseless case, which indicates the decrease in reconstruction quality. Since parameter β is closely associated with pupil update only, the three curves highly coincide with one another. The curves of parameter γ with three different values are quite similar, except that the curve γ = 1 is placed slightly below the other two. The simulation results above accounts for the suggested parameter ranges given in Table 2. Also, we have to admit that the demonstration is not strictly argued and may have certain contingency. For example, the ADMM update equations suggest that large values of the penalty parameter α tend to produce small prime residuals but simultaneously increase the values of dual residuals [41]. Proper

values for the parameters should be chosen to achieve balance for the convergence performance of the method are jointly determined by both residuals. The absolutely accurate ranges that guarantee the feasibility of ADMM method should be given after rigorous mathematical proof. In this paper, the suggested ranges for parameters have been proved to be effective in our simulations and experiments and can be used as reference.

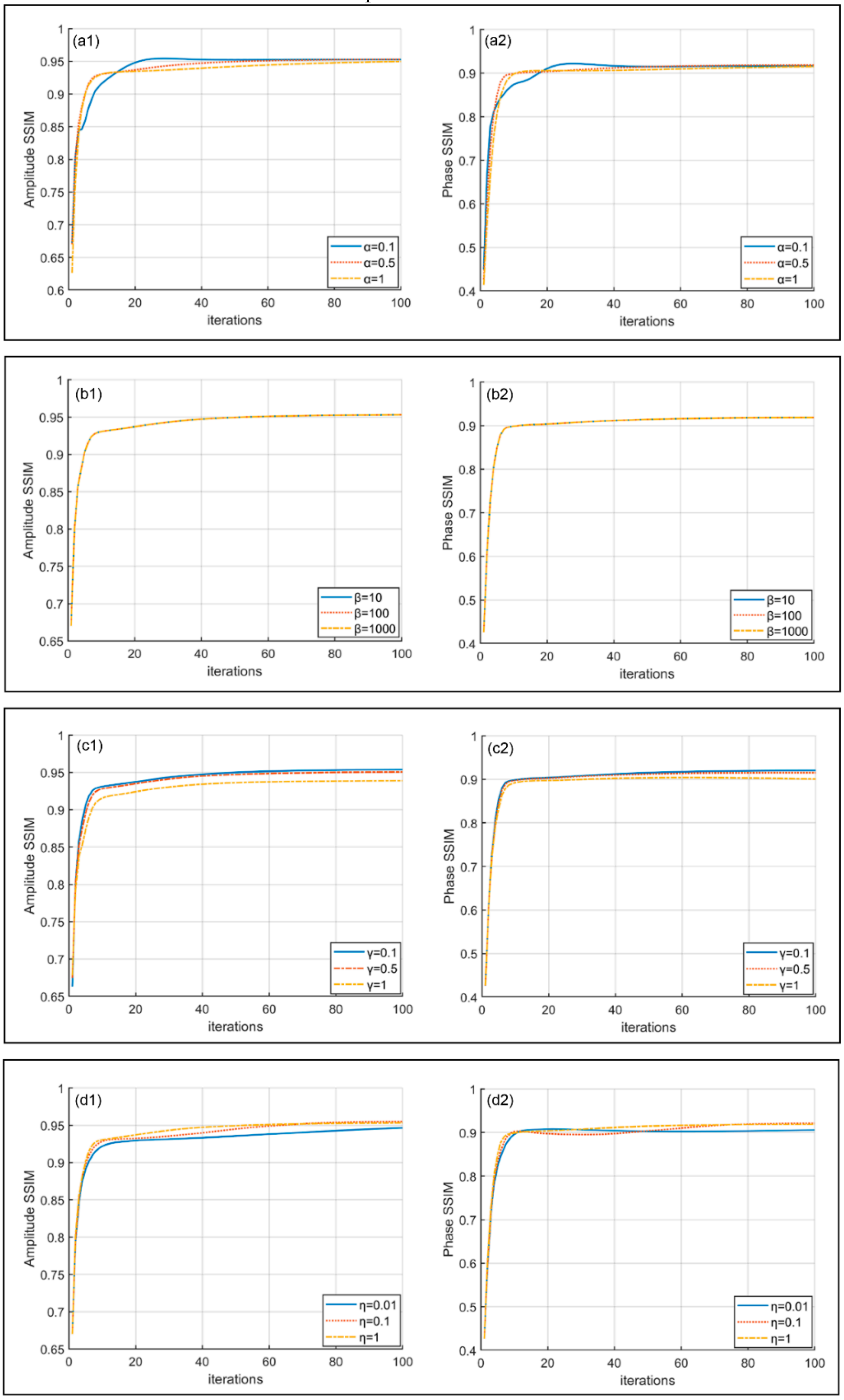

Figure S1. Comparison curves for different choices of parameters under noiseless conditions. For each parameter, three typical values are selected to examine how their variation affects the reconstruction results. (a1)-(a2) penalty parameter α. (b1)-(b2) regularization parameter β. (c1) –(c2) regularization parameter γ. (d1)-(d2) step size η.

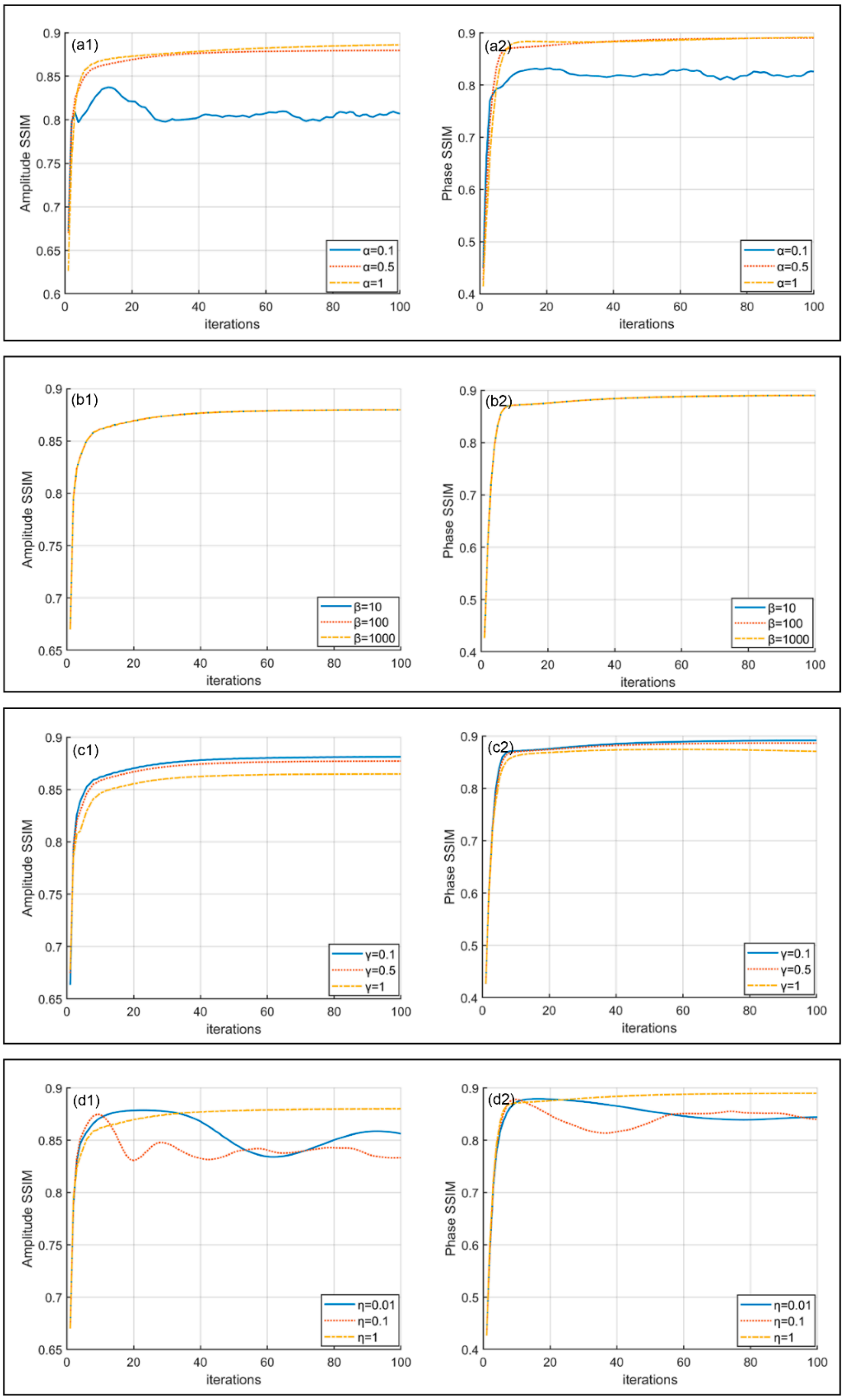

Figure S2. Comparison curves for different choices of parameters under 50% Gaussian noise. The graphs totally follow the organization of Fig. S1: (a1)-(a2) penalty parameter α. (b1)-(b2) regularization parameter β. (c1) –(c2) regularization parameter γ. (d1)-(d2) step size η.

Supplementary File S3. Discussions on self-adaptive step size strategy

Many extensions to the classic ADMM method have been reported and most of them rigorously present superior convergence performance in practical. One typical extension is to use different step sizes η^k for each iteration intended to make final results less dependent on the initial choice of step size. Though it can be difficult to prove the convergence of ADMM with varying step sizes, powerful analysis indicates that the original theory still applies if η becomes almost unchangeable after a finite

number of iterations. The framework of self-adaptive step size strategy for ADMM method is basically given as

$$\eta^{k+1} = \begin{cases} \tau\eta^k & R_p^k > \mu R_d^k \\ \eta^k / \tau & R_d^k > \mu R_p^k \\ \eta^k & otherwise \end{cases} \tag{S12}$$

where τ >1 and μ >1 are constant parameters. Choices of parameters that work well might be τ =2 and μ =10 [56]. The proposed scheme attempts to constrain primal and dual residuals within a certain range of each other when they both approaches zero.

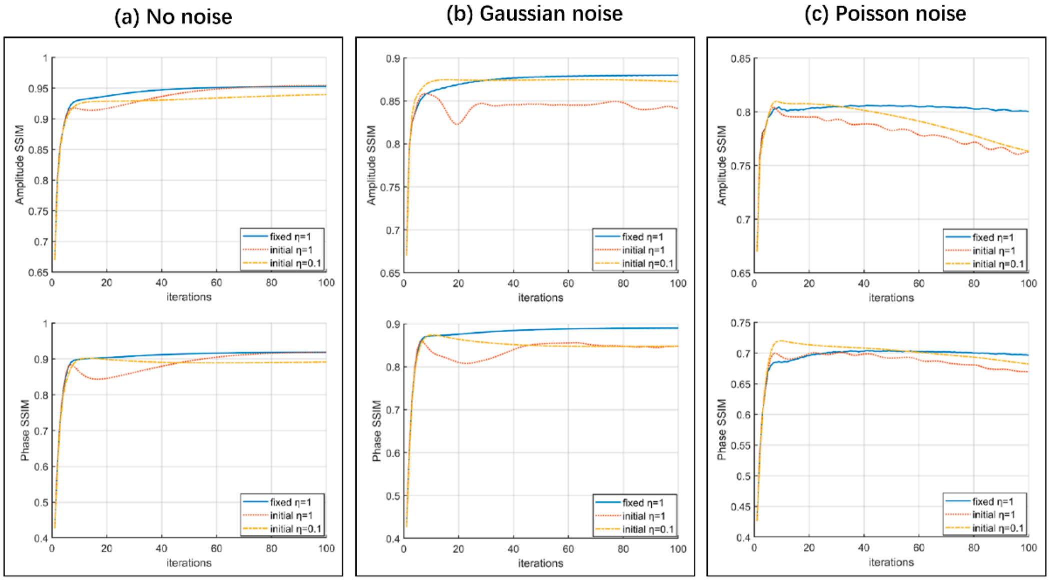

Figure S3. Comparison curves for our proposed method and other two schemes with self-adaptive step size update strategy under the conditions of: (a) no noise. (b) 50% Gaussian noise. (c) Poisson noise ($\sigma = 10^{-4}$).

We have set the suggested choice of step size as $\eta = 1$ in this paper. Here, we compare the reconstruction performance of FPM realized by three different ADMM schemes: (1) $\eta = 1$ without step size update; (2) self-adaptive step size with $\eta^0 = 1$; (3) self-adaptive step size with $\eta^0 = 0.1$. The introduction of $\eta = 0.1$ is inspired from the assumption that the initial step size should be small enough to stabilize computation and then increases accordingly to avoid stagnation of iterations. The comparison results under the condition of noise-free, Gaussian noise and Poisson noise are clearly shown in Fig. S3.

We find that all the three schemes perform without obvious differences during the first several iterations. As the iterations advance forward, the curves of scheme 2 present a state of fluctuation and especially violently with the disturbance of noise. The curves of scheme 3 shows a tendency of stable decrease, which achieves numerical results inferior to scheme 1. Generally speaking, the proposed method generates relatively better reconstruction results due to its high-quality convergence and stability despite its mild fluctuation under Poisson noise.

The validation seemingly contradicts the demonstration we made previously that the self-adaptive step size strategy outperforms the original ADMM method. This might be explained by the non-convex nature of FPM phase retrieval, as we always emphasize in this paper. On the other hand, the proposed modification is based on an essential theoretical prerequisite that both the primal residual and dual residual must converge to zero, which is difficult to achieve in practical FPM implementation.
